# Feasibility and Oncological Outcomes of Segmental Ureteral Resection Versus Radical Nephroureterectomy for High-Risk Ureteral Urothelial Carcinoma

**DOI:** 10.3390/cancers18152527

**Published:** 2026-08-06

**Authors:** Yu-Hsiang Chang, Chao-Hsiang Chang, Chi-Ping Huang, Wen-Jeng Wu, Ching-Chia Li, Marcelo Chen, Wun-Rong Lin, Chih-Chin Yu, Vincent F. S. Tsai, Yao-Chou Tsai

**Affiliations:** 1 Department of Urology, National Taiwan University Hospital, Taipei 100225, Taiwan; b01401068@gmail.com; 2 Department of Urology, China Medical University and Hospital, Taichung 404327, Taiwan; urology8395@yahoo.com.tw (C.-H.C.); huangchiping@yahoo.com.tw (C.-P.H.); 3 School of Medicine, China Medical University, Taichung 404328, Taiwan; 4 Department of Urology, Kaohsiung Medical University Hospital, Kaohsiung 807656, Taiwan; wejewu@kmu.edu.tw (W.-J.W.); ccli1010@hotmail.com (C.-C.L.); 5 Department of Urology, School of Medicine, College of Medicine, Kaohsiung Medical University, Kaohsiung 807378, Taiwan; 6 Graduate Institute of Clinical Medicine, College of Medicine, Kaohsiung Medical University, Kaohsiung 807378, Taiwan; 7 Department of Urology, MacKay Memorial Hospital, Taipei 104217, Taiwan; marcelo@mmh.org.tw (M.C.); vincent751051@gmail.com (W.-R.L.); 8 Department of Urology, School of Medicine, Mackay Medical College, Taipei 252005, Taiwan; 9 Mackay Junior College of Medicine, Nursing, and Management, Taipei 112601, Taiwan; 10 Division of Urology, Department of Surgery, Taipei Tzu Chi Hospital, The Buddhist Tzu Chi Medical Foundation, New Taipei City 231542, Taiwan; b91401049@gmail.com; 11 School of Medicine, Buddhist Tzu Chi University, Hualien 970374, Taiwan; 12 Department of Urology, Taipei Medical University Hospital, Taipei Medical University, Taipei 110404, Taiwan

**Keywords:** upper tract urothelial carcinoma, segmental ureterectomy, radical nephroureterectomy, kidney-sparing surgery, renal function preservation, overlap weighting, competing risks

## Abstract

**Background/Objectives**: Radical nephroureterectomy (RNU) is the standard of care for high-risk upper tract urothelial carcinoma (UTUC) but causes permanent renal decline, often disqualifying patients from essential cisplatin-based adjuvant chemotherapy. Segmental ureteral resection (SUR) preserves renal function, but its safety in high-risk patients remains fiercely debated due to historical treatment selection biases and a lack of competing risk adjustments. We aimed to compare long-term oncological outcomes and postoperative renal function preservation between SUR and RNU for high-risk UTUC strictly localized to the ureter. **Methods**: Retrospective data from 859 patients (783 RNU, 76 SUR) with high-risk ureteral UTUC (high-grade or pathologic T2–T4) were analyzed from a 21-hospital nationwide database. Propensity score overlap weighting was implemented to achieve covariate balance. Overall survival (OS) was assessed via Cox proportional hazards regression, whereas cancer-specific survival (CSS), metastasis-free survival (MFS), and local recurrence-free survival (LRFS) were evaluated using multivariable Fine–Gray subdistribution hazard models to robustly account for the competing risk of non-cancer mortality. **Results**: Overlap weighting achieved excellent baseline comparability with an effective sample size of 429.5 patients per cohort. Weighted analyses demonstrated comparable long-term trajectories between SUR and RNU for OS (*p* = 0.62), CSS (hazard ratio [HR]: 0.94, *p* = 0.835), and MFS (HR: 0.88, *p* = 0.664). The Fine–Gray model confirmed that the surgical approach was not a significant independent predictor of local recurrence (HR: 0.74, *p* = 0.351). Crucially, the SUR group demonstrated a significantly lower renal function decline both at 1 month (−0.11 vs. −10.58 mL/min/1.73 m^2^, *p* < 0.001) and through final clinical follow-up (−5.33 vs. −12.49 mL/min/1.73 m^2^, *p* = 0.001). **Conclusions**: For meticulously selected patients with high-risk ureteral UTUC, SUR provides equivalent oncological control and survival outcomes to standard RNU. Crucially, this kidney-sparing approach significantly preserves postoperative renal function, safeguarding the physiological reserve required for patients to maintain eligibility for optimal subsequent systemic adjuvant therapies.

## Simple Summary

Upper tract urothelial carcinoma is a rare cancer of the kidney’s drainage system. The standard treatment removes the entire kidney and ureter. While effective, this causes permanent kidney damage, often preventing patients from receiving life-saving chemotherapy later. This study investigated a kidney-sparing alternative—removing only the affected segment of the ureter—for patients with high-risk cancer. By analyzing data from a large national database using advanced statistics, we found that the kidney-sparing approach offers similar survival and cancer control rates to total removal. Crucially, patients preserved their kidney function significantly better. These findings prove that for selected patients, removing only the cancerous ureter is a safe alternative. This protects kidney health and ensures patients can tolerate future chemotherapy, providing strong evidence to update how clinicians manage this disease.

## 1. Introduction

Upper tract urothelial carcinoma (UTUC) is a relatively rare but significant malignancy, accounting for approximately 5% to 10% of all urothelial carcinomas. It demonstrates an estimated annual incidence of nearly two cases per 100,000 inhabitants in Western countries [1]. Current European Association of Urology (EAU) guidelines establish radical nephroureterectomy (RNU) as the standard of care for high-risk nonmetastatic disease [2,3]. Conversely, kidney-sparing surgery (KSS), including segmental ureterectomy, is primarily recommended for low-risk tumours to minimize the morbidity associated with renal function loss without compromising survival.

Although RNU provides robust oncological control, removing the entire renal unit inevitably causes a significant and irreversible decline in renal function. Multi-institutional studies have demonstrated that patients experience a permanent median estimated glomerular filtration rate (eGFR) decline of 25% to 32% (an absolute decrease of approximately 12–14 mL/min/1.73 m^2^) following RNU [4,5]. This deterioration often precludes a substantial proportion of patients from meeting the renal threshold required for adjuvant cisplatin-based chemotherapy, missing a critical therapeutic window to reduce distant metastasis risk. Consequently, segmental ureterectomy has emerged as a crucial alternative to preserve nephron mass. However, its oncological safety—particularly regarding its efficacy in local recurrence control compared to RNU—remains heavily debated. Furthermore, existing comparative studies are largely limited by small sample sizes, baseline selection bias, and the failure of conventional survival analyses (such as standard Cox or Kaplan–Meier methods) to account for competing risks like non-cancer mortality, potentially biasing local recurrence estimates.

To overcome these limitations and provide more definitive clinical evidence, well-adjusted statistical methodologies are essential. In this study, we applied an overlap weighting (OW) scheme based on propensity scores to mimic the attributes of a randomized controlled trial, achieving a rigorous balance of baseline clinical characteristics between treatment groups. Furthermore, a multivariable Fine–Gray hazard model was implemented to accurately evaluate recurrence outcomes by appropriately accounting for the competing risk of death. Consequently, this study aimed to comprehensively compare the oncological outcomes of patients presenting with high-risk UTUC strictly localized to the ureter who were managed with either RNU or a segmental approach. We specifically evaluated overall survival (OS), cancer-specific survival (CSS), metastasis-free survival (MFS), and local recurrence rates. Our findings demonstrate that SUR provides equivalent oncological control to RNU across all survival endpoints, while significantly preserving postoperative renal function. This highlights the feasibility of SUR as a safe alternative for meticulously selected high-risk patients to maintain their eligibility for subsequent adjuvant therapies.

## 2. Materials and Methods

### 2.1. Study Population

Data were extracted from the Taiwan UTUC Collaboration Database, a comprehensive multicenter registry comprising clinical data from 21 hospitals across Taiwan. For this comparative analysis, we retrospectively identified patients with high-risk ureteral urothelial carcinoma who underwent surgical management between July 1988 and May 2024. Inclusion criteria were restricted to patients with high-risk disease, defined as having high-grade histology or pathological stage T2–T4 (pT2–T4) tumors strictly localized to the ureter. Exclusion criteria included preoperative evidence of regional lymph node involvement (cN+) or distant metastases (cM1), the presence of concurrent tumors involving both the ureter and renal pelvis, or incomplete datasets for essential variables. The eligible cohort was categorized into two groups based on the surgical approach: the radical nephroureterectomy (RNU) group and the segmental ureteral resection (SUR) group. The study protocol adhered to the principles of the Declaration of Helsinki and was approved by the Institutional Review Board of Taipei Tzu Chi Hospital (no. 06-X34-105). The requirement for informed consent was waived due to the retrospective design of the study.

### 2.2. Definition of Variables and Oncological Outcomes

Baseline demographic and clinicopathological characteristics were systematically recorded. Patient performance status was assessed using the Eastern Cooperative Oncology Group (ECOG) score, and clinical and pathological tumor staging was determined according to the 2017 TNM classification system. Perioperative safety was evaluated by grading surgical complications according to the Clavien–Dindo classification. Regarding the preoperative diagnostic workup, baseline evaluations routinely involved cross-sectional imaging and urine cytology. However, owing to the retrospective and multicenter nature of the database, the specific method of urine collection was not uniformly standardized across all institutions. Consequently, the preoperative urine cytology evaluations analyzed in this study comprised a heterogeneous mix of both standard voided urine samples and endoscopic urine barbotage cytology obtained during intervention. We acknowledge this lack of granularity in differentiating the exact cytology source for each patient as an inherent limitation of our retrospective dataset. Furthermore, preoperative ureteroscopy was aggressively utilized to directly visualize the tumor, confirm the anatomical location, and obtain tissue biopsies. Specifically, 536 patients (68.5%) in the RNU group and 52 patients (68.4%) in the SUR group underwent preoperative ureteroscopic evaluation. It is important to emphasize that these procedures were strictly diagnostic in nature; no patients in this analyzed cohort underwent prior endoscopic tumor ablation before their definitive surgical resection.

The primary survival endpoints were overall survival (OS) and cancer-specific survival (CSS). OS was defined as the time from surgery to death from any cause, whereas CSS was calculated from the date of surgery to death directly attributable to cancer. The specific cause of death was ascertained via official death certificates. Secondary endpoints encompassed postoperative renal function preservation, metastasis-free survival (MFS), and local recurrence-free survival (LRFS). Specifically, MFS was defined as the time from surgery to the first documented distant metastasis. LRFS was calculated as the time from surgery to tumor recurrence within the operative bed. Disease recurrence or metastasis was confirmed through imaging studies or pathological biopsy. Finally, renal function preservation was assessed by calculating the absolute change in the estimated glomerular filtration rate (∆ eGFR) at 1 month postoperatively and at the final clinical follow-up, with eGFR calculated using the Chronic Kidney Disease Epidemiology Collaboration (CKD-EPI) equation.

### 2.3. Postoperative Surveillance

Following surgery, standard surveillance protocols included a physical examination, urine cytology, renal ultrasonography, and cystoscopy at intervals of 3 to 6 months. Routine cross-sectional imaging was performed every 6 to 12 months to monitor for disease recurrence. When clinically indicated, further evaluations such as whole-body bone scans and chest computed tomography were conducted.

### 2.4. Statistical Analysis

Baseline patient characteristics were summarized using means and standard deviations for continuous variables, whereas categorical data were reported as frequencies and proportions. The absolute standardized mean difference (SMD) was utilized to evaluate the balance of covariates between the RNU and SUR groups. Covariates exhibiting an unweighted SMD greater than 0.1 were considered imbalanced. To rigorously adjust for treatment selection bias and account for these baseline discrepancies, propensity score (PS) overlap weighting was implemented.

**Figure 1:**
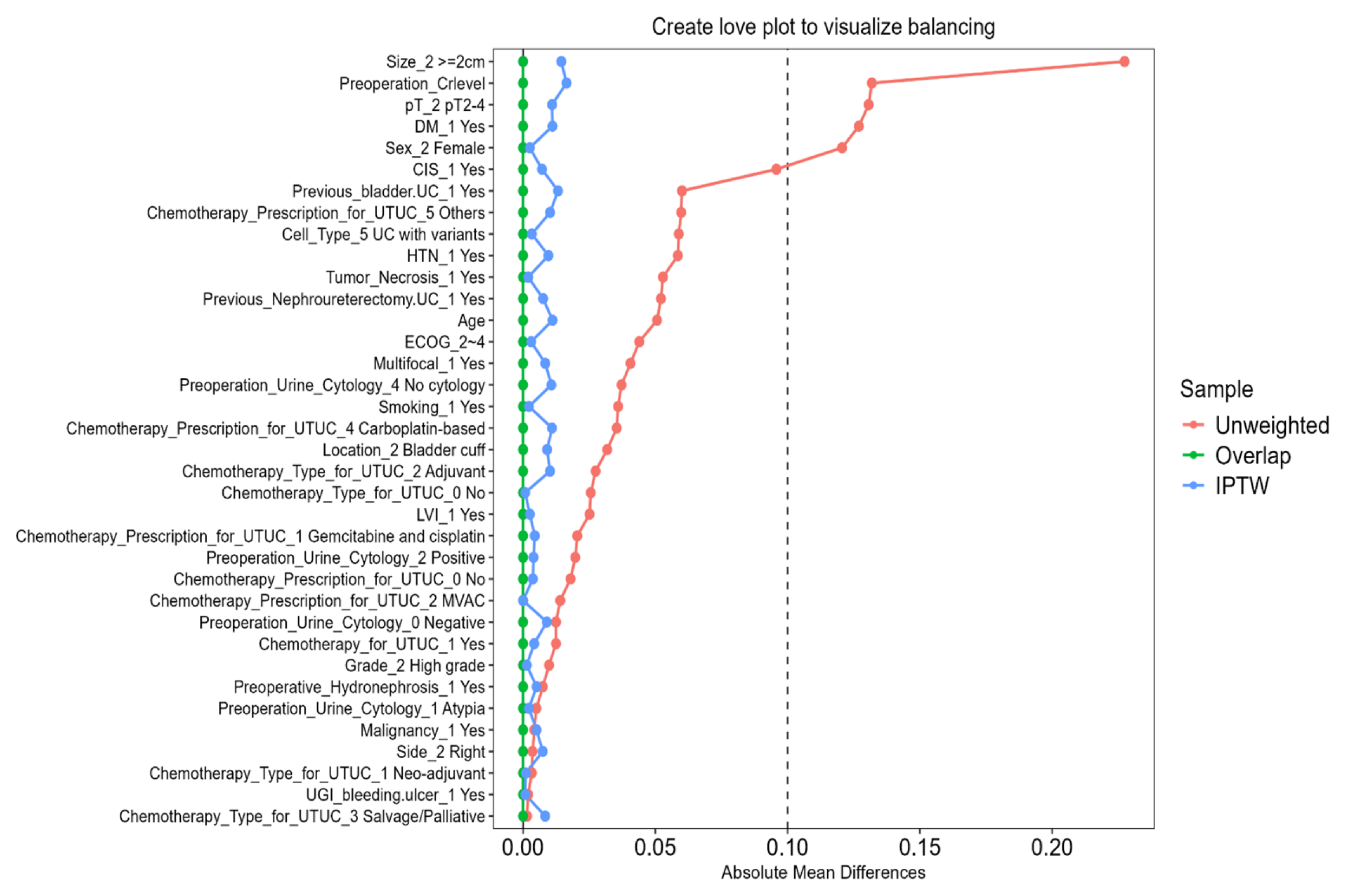
Love plot between unweighted and weighted.

A multivariable logistic regression model was employed to estimate the PS for each patient, incorporating potential confounders and imbalanced clinical factors, including age, sex, ECOG performance status, baseline renal function, and tumor characteristics. Within the overlap weighting framework, patients who underwent SUR (the treated cohort) were assigned a weight of 1 minus their PS, while those who underwent RNU (the untreated cohort) received a weight equal to their PS. This approach effectively minimizes the influence of patients at the extremes of the probability spectrum for either treatment, while amplifying the statistical weight of patients with clinical equipoise (i.e., comparable treatment probabilities). Consequently, this method mimics the covariate balance typically achieved in randomized controlled trials. A Love plot was generated to visually validate this balance, confirming that overlap weighting yielded superior baseline comparability between the SUR and RNU cohorts compared to the conventional inverse probability of treatment weighting (IPTW) method (Figure 1).

Subsequent survival analyses were conducted on this overlap-weighted cohort. For the assessment of OS, patients alive at the final follow-up were censored. To properly address biases introduced by competing events, death from non-cancer-related causes was defined as a competing risk for CSS, MFS, and LRFS.

Survival differences and prognostic indicators for OS were evaluated using univariable and multivariable Cox proportional hazards regression models. Variables demonstrating statistical significance in the univariable analysis were subsequently incorporated into the multivariable models. For oncological endpoints involving competing risks (CSS, MFS, and LRFS), univariable and multivariable Fine–Gray subdistribution hazard models were utilized. Specifically, potential prognostic indicators were initially evaluated via univariable Fine–Gray regression. To construct a rigorous and clinically meaningful multivariable model without overfitting, the primary variable of interest (surgical approach) was forced into the model, alongside covariates that demonstrated statistical significance (*p* < 0.05) in the univariable analysis across these competing-risk endpoints. Consequently, the final multivariable Fine–Gray subdistribution hazard models were consistently adjusted for a defined set of five specific factors: the surgical approach (RNU versus SUR), patient sex (male versus female), the presence of any prior systemic malignancy, smoking status, and a history of previous bladder urothelial carcinoma. All statistical tests were two-tailed, and a *p* value of <0.05 was considered statistically significant. All analytical procedures were executed using R (version 4.4.0) software (R Foundation for Statistical Computing, Vienna, Austria).

## 3. Results

### 3.1. Baseline Characteristics

A total of 859 patients were included in the analysis, with 783 receiving radical nephroureterectomy (RNU) and 76 receiving segmental ureteral resection (SUR). The mean age was 69.48 years in the RNU group and 69.00 years in the SUR group. The mean follow-up duration for overall survival was 57.50 months in the RNU group and 63.79 months in the SUR group. Table 1 presents the differences in baseline characteristics between the two groups before and after overlap weighting. Prior to overlap weighting, patients treated with SUR demonstrated a higher proportion of smaller tumor sizes (<2 cm) and lower pathological T stages. Furthermore, the SUR group had a higher incidence of diabetes mellitus and previous nephroureterectomy. After overlap weighting, all baseline covariates achieved excellent balance between the two groups, resulting in an effective sample size of 429.5 in each cohort.

### 3.2. Survival and Oncological Outcomes

#### 3.2.1. Overall Survival (OS)

**Figure 2:**
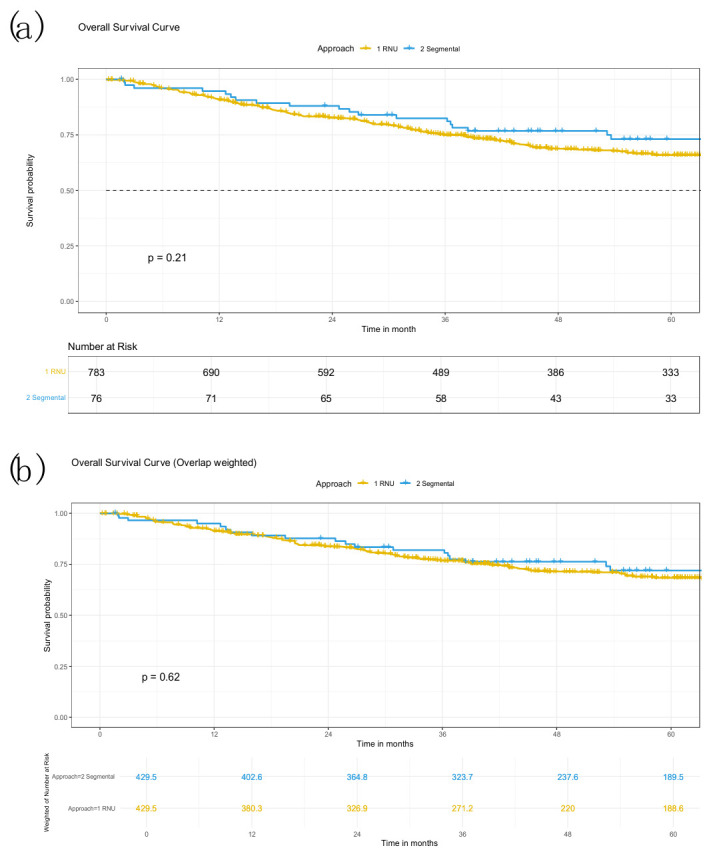
Overall survival curves: (**a**) overall survival curve; (**b**) overall survival curve (overlap-weighted).

In the unweighted cohort, both univariable (hazard ratio [HR]: 0.82, 95% confidence interval [CI]: 0.53–1.25, *p* = 0.35) and multivariable (HR: 0.86, 95% CI: 0.57–1.29, *p* = 0.453) Cox proportional hazards regression models demonstrated no significant difference in overall survival between the RNU and SUR groups (Table 2 and Table 3). Consistent with these statistical models, the unweighted Kaplan–Meier survival analysis also showed comparable OS trajectories (log-rank *p* = 0.21) (Figure 2a). To adjust for baseline confounders, overlap weighting was applied. Definitive analysis within the overlap-weighted cohort confirmed that OS remained equivalent between the two surgical approaches, as visually demonstrated by the closely aligned survival curves and significantly overlapping 95% confidence interval bands (weighted log-rank *p* = 0.62) (Figure 2b).

#### 3.2.2. Cancer-Specific Survival (CSS)

**Figure 3:**
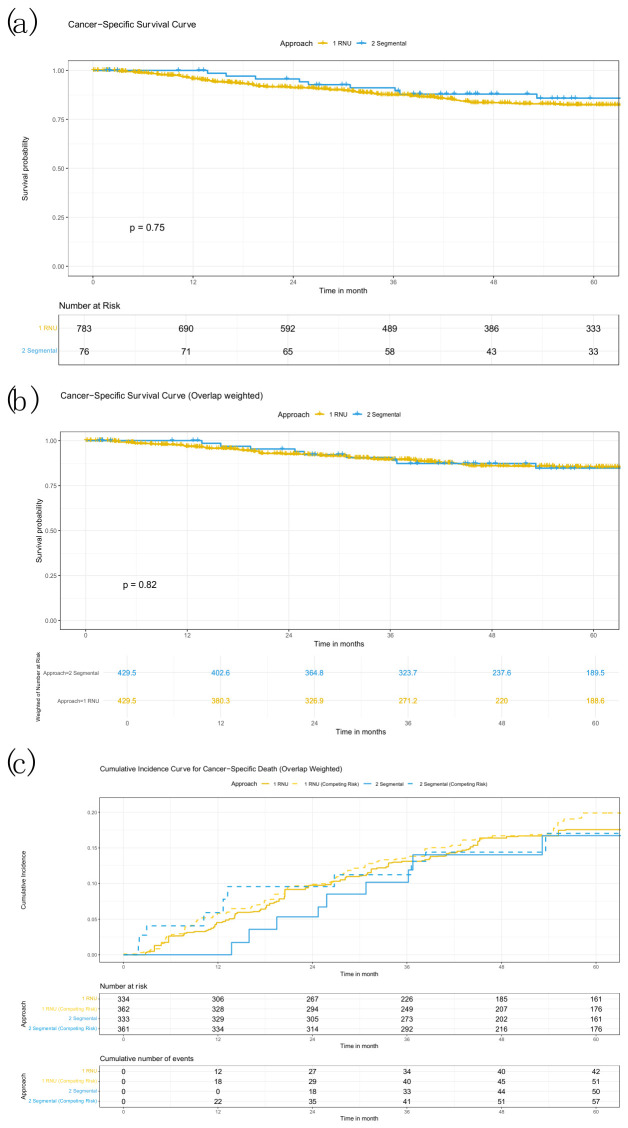
Cancer-specific survival and cumulative incidence: (**a**) cancer-specific survival curve; (**b**) cancer-specific survival curve (overlap-weighted); (**c**) cumulative incidence curves.

Prior to overlap weighting, cancer-specific survival (CSS) was comparable between the RNU and SUR groups, as demonstrated by Kaplan–Meier analysis (log-rank *p* = 0.75) (Figure 3a) and both univariable (HR: 1.10, 95% CI: 0.60–2.04, *p* = 0.75) and multivariable (HR: 1.22, 95% CI: 0.65–2.31, *p* = 0.536) Cox models (Table 2 and Table 3). In the weighted cohort, overlap-weighted Kaplan–Meier analysis revealed comparable CSS trajectories (weighted log-rank *p* = 0.82) (Figure 3b). Furthermore, cumulative incidence curves and the multivariable Fine–Gray model confirmed that SUR did not compromise CSS compared to standard RNU (HR: 0.94, 95% CI: 0.52–1.70, *p* = 0.835) (Figure 3c, Table 4).

#### 3.2.3. Metastasis-Free Survival (MFS)

**Figure 4:**
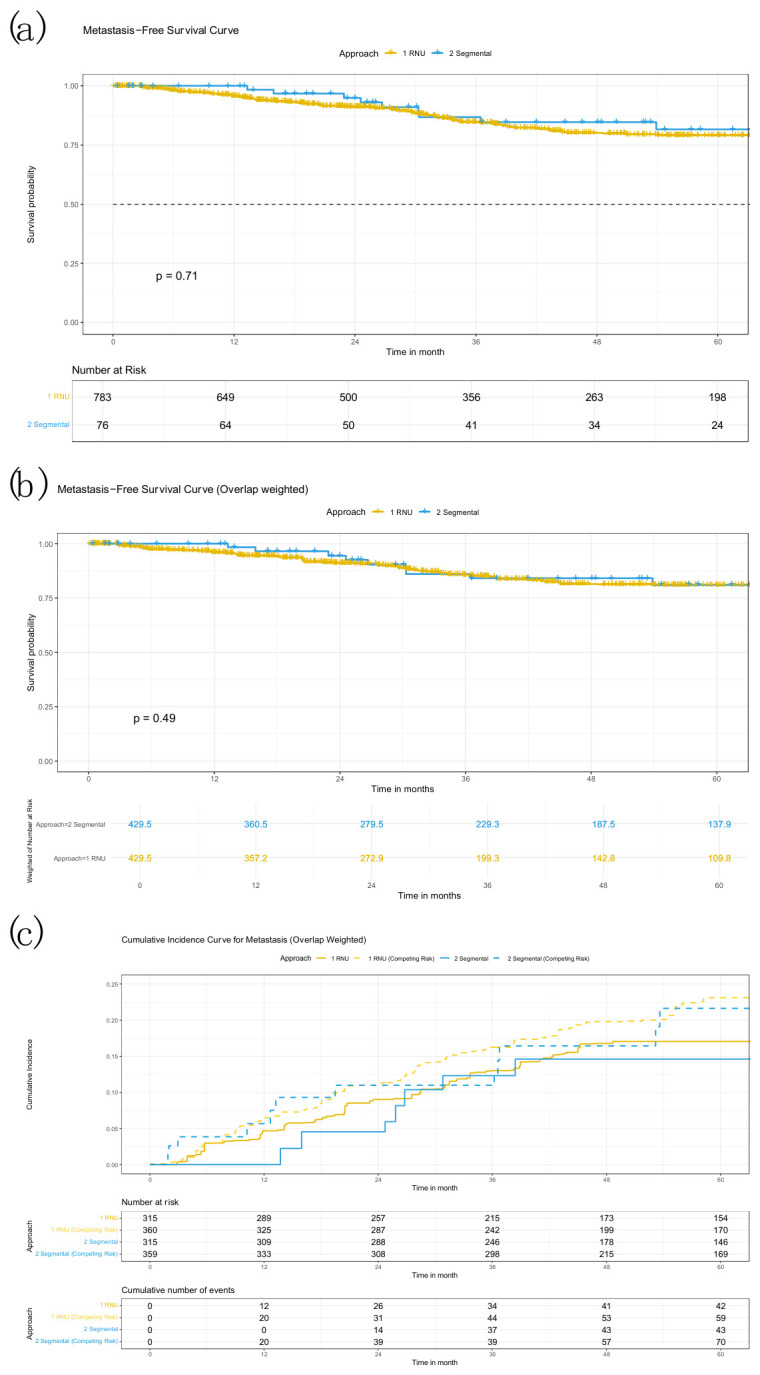
Metastasis-free survival and cumulative incidence: (**a**) metastasis-free survival curve; (**b**) metastasis-free survival curve (overlap-weighted); (**c**) cumulative incidence curves for metastasis.

Initial analysis of the unadjusted data revealed that metastasis-free survival (MFS) was comparable between the RNU and SUR groups, as demonstrated by Kaplan–Meier analysis (log-rank *p* = 0.71) and both univariable (HR: 0.92, 95% CI: 0.50–1.70, *p* = 0.796) and multivariable (HR: 0.97, 95% CI: 0.53–1.74, *p* = 0.909) Cox models (Figure 4a, Table 5 and Table 6). In the weighted cohort, overlap-weighted Kaplan–Meier analysis revealed comparable MFS trajectories (weighted log-rank *p* = 0.49) (Figure 4b). Furthermore, cumulative incidence curves and the multivariable Fine–Gray model confirmed that SUR did not significantly increase the risk of distant metastasis compared to standard RNU (HR: 0.88, 95% CI: 0.48–1.59, *p* = 0.664) (Figure 4c, Table 4).

#### 3.2.4. Local Recurrence and Local Recurrence-Free Survival (LRFS)

**Figure 5:**
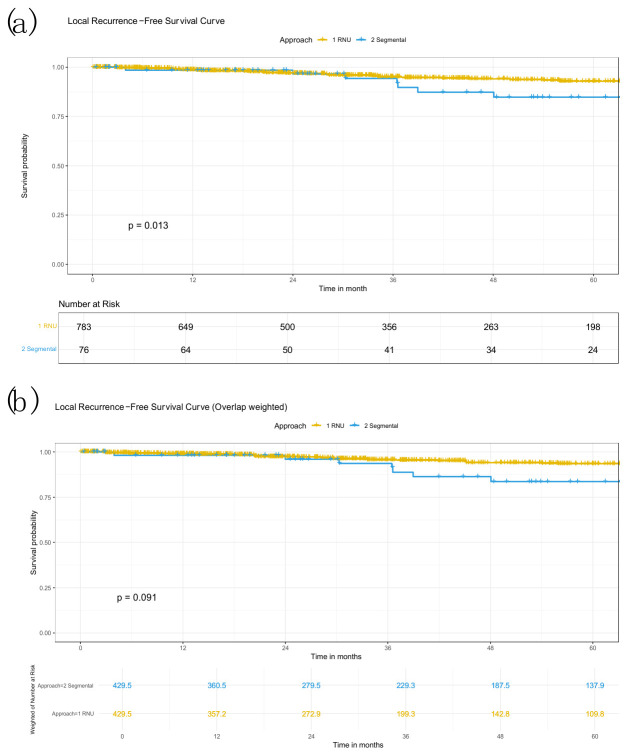
Local recurrence-free survival curves: (**a**) local recurrence-free survival curve; (**b**) local recurrence-free survival curve (overlap-weighted).

Regarding local recurrence, the SUR approach yielded a significantly higher recurrence rate than RNU, both before (13.2% vs. 5.1%, *p* = 0.009) and after overlap weighting adjustment (13.2% vs. 4.6%, *p* = 0.004) (Table 7). Multivariable Cox regression analysis further demonstrated that the surgical modality was an independent predictor of local recurrence-free survival (LRFS), with patients in the SUR group facing a significantly elevated risk of local recurrence relative to the RNU group (HR: 4.34, 95% CI: 1.60–11.77, *p* = 0.004) (Table 6). Kaplan–Meier survival analysis of the unweighted cohort indicated a significantly worse LRFS for patients undergoing the SUR approach (*p* = 0.013) (Figure 5a). However, following overlap weighting adjustment, the Kaplan–Meier curves revealed no statistically significant difference in LRFS between the two surgical modalities (*p* = 0.091) (Figure 5b). Consistent with the weighted Kaplan–Meier analysis, the Fine–Gray competing risk model demonstrated that the surgical approach was not a significant independent predictor of LRFS; the hazard of local recurrence for the SUR group was comparable to that of the RNU group (HR: 0.74, 95% CI: 0.39–1.40, *p* = 0.351) (Table 4). It is important to clarify that while Table 7 indicates the absolute percentage of local recurrence remains statistically higher in the SUR group even after overlap weighting, this persistent difference in raw unadjusted weighted percentages primarily reflects the high-risk baseline biology of the cohort and a longer exposure time to recurrence risk. It does not reflect the independent hazard of the surgery itself, which is definitively neutralized once competing non-cancer mortality risks and multivariable baseline adjustments are rigorously taken into account within the Fine–Gray model.

#### 3.2.5. Surgical Margin Status

Regarding the completeness of tumor excision, the positive surgical margin (PSM) rates were evaluated. In our study cohort, the surgical margin positivity rates were comparably low between the two surgical modalities. Specifically, a positive surgical margin was observed in 5.4% (42/783) of patients in the RNU group and 7.9% (6/76) of patients in the SUR group. There was no statistically significant difference in the risk of positive surgical margins between the kidney-sparing approach and radical nephroureterectomy in the unweighted cohort (*p* = 0.512) (Table 7).

### 3.3. Risk Factors Associated with Oncological Outcomes

In multivariable analyses, worse OS was predicted by older age (HR: 1.05, *p* < 0.001), ECOG 2–4 (HR: 2.49, *p* = 0.003), multifocality (HR: 1.89, *p* = 0.018), and pT2–4 (HR: 1.80, *p* = 0.008) (Table 3). Independent risk factors for poorer CSS included ECOG 2–4 (HR: 3.76, *p* = 0.023), pT2–4 (HR: 2.54, *p* = 0.036), LVI (HR: 2.21, *p* = 0.036), and salvage/palliative chemotherapy (HR: 2.84, *p* = 0.013) (Table 3), as well as prior malignancies (HR: 1.56, *p* = 0.041) (Table 4). Elevated local recurrence risk correlated with hypertension (HR: 3.25, *p* = 0.028), pT2–4 (HR: 3.57, *p* = 0.010), higher creatinine (HR: 1.37, *p* < 0.001), and carboplatin (HR: 5.53, *p* = 0.042) (Table 6). Worse MFS was driven by high tumor grade (HR: 11.35, *p* = 0.035), LVI (HR: 3.65, *p* = 0.002), and chemotherapy (*p* ≤ 0.003) (Table 6). Conversely, female sex demonstrated a strong protective effect against both mortality (HR: 0.52, *p* = 0.009) (Table 3) and local recurrence (HR: 0.58, *p* = 0.001) (Table 4).

### 3.4. Renal Function Preservation

The preservation of postoperative renal function was significantly superior in the SUR group. While preoperative estimated glomerular filtration rate (eGFR) was comparable between the weighted groups (RNU: 49.91 vs. SUR: 46.49, *p* = 0.247), the SUR group demonstrated a significantly lower absolute decline in eGFR (∆eGFR) at 1 month postoperatively (−0.11 vs. −10.58, *p* < 0.001) (Table 7). To further illustrate these functional dynamics, we analyzed shifts in CKD stages and their direct impact on eligibility for cisplatin-based chemotherapy (defined as eGFR > 60 mL/min/1.73 m^2^). Preoperatively, both CKD stage distribution and cisplatin eligibility were comparable between the RNU and SUR cohorts (eligibility: 27.7% vs. 26.3%, *p* = 0.900) (Table S1). However, at one month postoperatively, the RNU group experienced a significant downward shift in renal function staging (*p* < 0.001), causing the proportion of cisplatin-eligible patients to plummet to 16.5%. In contrast, the SUR group effectively mitigated this stage progression, robustly maintaining chemotherapy eligibility at 25.0% (Table S1). This protective benefit was sustained until the last clinical follow-up, where the decline in eGFR remained significantly less severe in patients undergoing SUR compared to those treated with RNU (−5.33 vs. −12.49, *p* = 0.001) (Table 7).

## 4. Discussion

Our multicenter study demonstrates that for patients with high-risk ureteral UTUC, segmental ureteral resection (SUR) achieves oncological outcomes comparable to those of radical nephroureterectomy (RNU). By utilizing propensity score overlap weighting to ensure a robust balance of baseline covariates, our analysis confirmed no significant differences between the two surgical approaches in terms of overall survival (OS), cancer-specific survival (CSS), and metastasis-free survival (MFS). Most importantly, the preservation of postoperative renal function was significantly superior in the SUR group, exhibiting a markedly lower decline in eGFR at both one month postoperatively and through the final clinical follow-up.

This renal protective benefit translates into a critical clinical advantage, particularly regarding the feasibility of systemic therapy. While cisplatin-based adjuvant chemotherapy (AC) remains a cornerstone in optimizing long-term prognosis for high-risk UTUC, its full-dose administration strictly requires an eGFR > 60 mL/min/1.73 m^2^. Given that this aging, comorbid population frequently harbors baseline renal insufficiency, preserving every unit of renal function is paramount. Multiple international and Asian cohorts have consistently confirmed this global trend, demonstrating that RNU significantly reduces postoperative eGFR and frequently plummets cisplatin eligibility to under 15–25% [4,6,7,8]. Consequently, this RNU-induced renal decline often irrevocably disqualifies vulnerable patients from receiving life-prolonging treatments.

In our real-world cohort, the actual administration rates of cisplatin-based regimens were comparable between the SUR and RNU groups (13.2% vs. 11.1%, *p* = 0.322). While this equivalence likely reflects the multifactorial nature of retrospective clinical decision-making (e.g., patient preference, physician bias and institutional protocols), postoperative renal function dynamics powerfully quantifies the underlying physiological benefit of SUR. When evaluating patients with baseline CKD stages 2, 3, and 4, the RNU cohort exhibited a pronounced stage deterioration at one month postoperatively. This downward migration was characterized by a marked depletion of early-stage patients and a significant increase in progression to end-stage renal disease (CKD Stage 5 increased from 15.3% to 20.1%). In stark contrast, the SUR group demonstrated a robust protective effect against CKD progression, stabilizing stage migration and effectively preventing the irreversible renal decline observed in the radical cohort. Consequently, this protective effect directly translated into the safeguarding of systemic therapy options: while preoperative cisplatin eligibility (eGFR > 60) was comparable, the RNU cohort saw this eligibility plummet to 16.5% postoperatively, whereas SUR actively maintained this critical physiological reserve at 25.0%. The magnitude of renal function preservation observed with SUR—providing a mean eGFR advantage of 12–17 mL/min/1.73 m^2^ over RNU [9]—acts as a critical prerequisite. By effectively minimizing surgical nephron loss, SUR actively safeguards the physiological reserve necessary for patients to maintain this strict eGFR threshold and safely tolerate optimal systemic therapies when clinically indicated.

The optimal surgical management for high-risk UTUC remains debated. The 2025 EAU guidelines establish RNU as the standard of care for high-risk disease, recommending kidney-sparing surgery (KSS) primarily for low-risk cases. Under the EAU framework, performing segmental ureterectomy (SU) for high-risk UTUC is considered only in highly selected patients with imperative indications (e.g., solitary kidney or severe renal insufficiency), reflecting the limited evidence and a cautious stance on the risk of disease progression [2,3]. Conversely, the 2023 AUA/SUO and NCCN (v1.2026) guidelines adopt a more flexible approach, recognizing SU as an appropriate option for carefully selected, surgically eligible high-risk tumors, particularly those confined to the distal or mid-ureter [1,10]. Amidst these diverging recommendations, our multicenter, overlap-weighted analysis provides complementary real-world evidence supporting the oncological safety of SUR in selected patients, resonating with the cautious but evolving landscape of current guidelines.

Notably, our study cohort provides a comprehensive anatomical representation: while 60% of the tumors were located in the lower ureter, a substantial 40% were situated in the mid or upper ureter. By incorporating this broader anatomical distribution, our analysis demonstrates that for high-risk tumors strictly localized to the ureter—regardless of the specific segment—SUR achieves survival outcomes entirely comparable to RNU. Specifically, we observed no significant differences in OS (*p* = 0.62), CSS (HR: 0.94, *p* = 0.835), or MFS (HR: 0.88, *p* = 0.664). These findings are particularly noteworthy given that the NCCN Principles of Surgical Management identify mid- and proximal ureteral tumor locations as less favorable criteria for nephron preservation due to technical challenges, and a prospective international registry study (CROES-UTUC) reported that proximal ureteral UTUC had worse OS and CSS outcomes than other tumor locations following segmental resection [10,11]. Our results suggest that with meticulous patient selection and appropriate surgical technique, the anatomical limitations traditionally attributed to non-distal ureteral tumors may be successfully overcome. These results are highly consistent with recent propensity-matched cohorts and hospital-based registries, which demonstrate equivalent survival outcomes and superior renal preservation for SUR compared to RNU [12,13]. Notably, this oncological equivalence persists even among patient subgroups with high-grade or advanced-stage (T2-4/N+) distal ureteral tumors [14,15,16]. Importantly, these findings are corroborated by European multicenter experiences demonstrating the technical feasibility and comparable oncological safety of distal segmental ureterectomy [17]. Furthermore, subsequent analyses by the same group confirmed that SUR achieves equivalent survival while critically mitigating renal decline in patients with preoperative impairment, providing essential foundational support for our large-scale findings [18]. Ultimately, our results help contextualize the EAU’s concerns regarding disease progression, providing critical evidence that when patient selection is meticulously conducted, SUR can be a safe and effective alternative to RNU.

A critical and often debated nuance in the kidney-sparing management of UTUC pertains to the risk of local recurrence. While several studies have identified SUR as a predictor of reduced recurrence-free survival (RFS) compared to RNU [16]—for instance, a comparative study by Guan et al. reported a significantly higher local recurrence rate for segmental resection on Kaplan–Meier analysis (*p* < 0.001) and identified the kidney-sparing approach as a strong independent risk factor in standard multivariable Cox regression (HR: 4.26, *p* < 0.001) [19]. Moreover, an analysis of local recurrence patterns following SUR by the same group highlighted a considerable absolute risk, reporting 3-year rates of local recurrence (31.6%), lymph node metastasis (19.0%), and ipsilateral upper urinary tract recurrence (22.2%), culminating in an overall local recurrence rate of 39% [20]. Our initial unadjusted analysis perfectly mirrored this trend, showing a significantly higher local recurrence rate in the SUR cohort (13.2% vs. 5.1%, *p* = 0.009). However, this disparity underscores the profound impact of inherent treatment selection biases—such as baseline imbalances in pathological stage and grade—and competing events. These are major methodological concerns explicitly acknowledged in the EAU-commissioned systematic review [3]. To rigorously address these confounders, we applied propensity score overlap weighting, after which the Kaplan–Meier trajectories for local recurrence-free survival (LRFS) demonstrated no statistically significant difference between the two surgical modalities (*p* = 0.45). Furthermore, our multivariable Fine–Gray competing risk model definitively confirmed that the surgical approach itself is not an independent predictor of local recurrence (HR = 0.74, *p* = 0.351). As observed in our results, the apparent persistence of a higher absolute local recurrence percentage in the weighted SUR cohort merely reflects the complex interplay of high-risk baseline tumor biology and longer exposure intervals, rather than an inherent oncological deficiency of the surgical procedure itself. The fundamental discrepancy between the Cox regression and the Fine–Gray analysis stems from how each statistical method handles competing events, such as non-cancer mortality. Standard Cox proportional hazards regression censors patients who die from competing causes, implicitly and incorrectly assuming they remain at risk for local recurrence. In an aging cohort with associated comorbidities, this right-censoring mathematically overestimates the true clinical risk of local recurrence. In contrast, the Fine–Gray subdistribution hazard model accurately accounts for these competing events by maintaining them in the risk set, thereby reflecting a more realistic and clinically relevant probability of actual recurrence. Given the high likelihood of competing mortality in this specific patient population, we consider the Fine–Gray model to be the primary and definitive analysis for interpreting local recurrence risks. Notably, this highlights the methodological advantage of addressing competing non-cancer mortality; had we relied solely on standard multivariable Cox regression, segmental resection would have falsely appeared as a significant risk factor for local recurrence (HR = 4.34, *p* = 0.004, as shown in our unadjusted multivariable Cox analysis)—a magnitude of risk remarkably identical to the HR of 4.26 reported by Guan et al. [19]. Instead, intrinsic tumor characteristics are the true drivers of oncological outcomes. This is robustly supported by contemporary SU-specific cohorts. Specifically, larger tumor size (≥2 cm) and pathological stage ≥ T2 were significant prognostic factors of poor urothelial RFS (all *p* < 0.05) [21]. Furthermore, Guan et al. explicitly identified high tumor grade (G3) as a strong independent risk factor for both local recurrence (HR = 3.355, 95% CI 1.375–8.184, *p* = 0.008) and lymph node metastasis (HR = 3.782, 95% CI 1.036–13.812, *p* = 0.044), alongside lymphovascular invasion (LVI) [20]. These findings strongly suggest that historically reported elevated recurrence rates associated with SUR are largely artifacts of confounding clinical variables and competing risks. Nevertheless, as emphasized by Guan et al., while SU is a feasible treatment for selected patients, the inherent risks associated with these adverse pathological features dictate that careful postoperative monitoring and active adjuvant therapy remain essential to minimize recurrence [20]. This conclusion is highly consistent with propensity-matched analyses from the CROES-UTUC international registry and other robust cohorts, which demonstrate comparable RFS, PFS, and MFS between the two modalities following appropriate statistical adjustment [11,13].

A fundamental oncological concern frequently raised regarding SUR is the heightened risk of positive surgical margins (PSM). Extensive literature demonstrates that SUR cohorts exhibit significantly higher PSM rates compared to their RNU counterparts. This disparity primarily stems from the anatomical constraints of achieving wide excisional boundaries while preserving adequate ureteral length for reconstruction. A recent study by Lima et al. strikingly illustrated this vulnerability, revealing that the PSM rate in segmental procedures performed without intraoperative frozen section analysis (FSA) reached as high as 42.9%; in stark contrast, RNU inherently affords wider excision margins and a correspondingly lower PSM risk [22].

The clinical impact of margin positivity is substantial, as extensive literature—including large cohorts from the Cleveland Clinic and Colin et al.—consistently confirms that PSM is a potent, independent predictor for significantly worse overall, cancer-specific, and metastasis-free survival [23,24]. This inherent surgical challenge highlights the delicate balance between nephron preservation and complete tumor extirpation mandated by current guidelines [1]. Importantly, in our present cohort, the actual PSM rate for SUR was 7.9%, which was not significantly higher than the 5.4% observed in the RNU group (*p* = 0.512). This comparable margin status further supports the oncological safety of SUR when performed in meticulously selected patients.

Beyond the surgical modality, our multivariable analyses confirmed that inherent clinical and pathological characteristics are the true drivers of UTUC prognosis. Consistent with established systematic reviews [25], advanced age, poorer performance status (ECOG 2–4), higher pathological stage (pT2–4), tumor multifocality, and lymphovascular invasion (LVI) were robustly associated with worse survival endpoints. These findings validate the reliability of our multicenter cohort and underscore the aggressive biological nature of advanced UTUC. Notably, our analysis revealed two clinically significant insights. First, female sex demonstrated a striking protective effect, independently correlating with a reduced risk of both overall mortality (HR: 0.52, *p* = 0.009) and local recurrence (HR: 0.58, *p* = 0.001). While female sex demonstrated a strong protective effect in our cohort—a finding corroborated by other Asian multicenter studies [26]—its prognostic role in UTUC remains highly debated and appears heavily influenced by regional or ethnic differences [27,28]. Second, elevated preoperative creatinine levels were significantly associated with an increased risk of local recurrence in our cohort (HR: 1.37, *p* < 0.001).

This finding is robustly supported by several original studies emphasizing the prognostic value of baseline renal function. For instance, Ito et al. identified a preoperative eGFR < 60 mL/min/1.73 m^2^ as a strong independent predictor of extraurothelial recurrence following RNU [29]. Similarly, a multicenter analysis by Freifeld et al. focusing on high-grade UTUC demonstrated that a baseline eGFR ≥ 50 mL/min/1.73 m^2^ significantly correlates with a lower risk of disease recurrence (HR = 0.48, *p* = 0.028) [30]. Furthermore, Muramoto et al. recently confirmed that preoperative chronic kidney disease (CKD) independently predicts worse non-urothelial recurrence-free, cancer-specific, and overall survival [31].

The clinical implications of this baseline vulnerability are profound when considering the surgical impact of RNU. In their large multicenter cohort, Muramoto et al. observed a severe median eGFR decline from 54.7 to 40.6 mL/min/1.73 m^2^ post-RNU, with a mere 5.1% of patients maintaining an eGFR ≥ 60 [31]. This drastic reduction compounding on preexisting renal fragility highlights a critical issue: nearly three-quarters of patients develop surgically induced CKD (stage ≥ 3a) following RNU, which is a well-established risk factor for worsened all-cause mortality (HR 1.42, *p* = 0.032 for CKD-S3b) [32].

Highlighting the extreme consequence of this renal functional decline, a recent study by Lee et al. demonstrated that patients rendered dialysis-dependent after RNU suffer dramatically worse overall survival (3-year OS: 29% vs. 60%, HR 2.13, *p* = 0.03), despite exhibiting oncologic outcomes comparable to non-dialysis patients [33]. Collectively, these findings reinforce our primary thesis: actively preserving renal function through kidney-sparing surgery is critical not only for maintaining eligibility for adjuvant cisplatin-based chemotherapy but is also intrinsically linked to mitigating non-cancer mortality and achieving superior overall disease control.

This study has several distinct strengths that address the methodological gaps of prior investigations. While previous multicenter cohorts have provided valuable insights, they predominantly relied on propensity score matching, which inherently discards unmatched patient data and reduces statistical power [16,34]. In contrast, our 21-hospital nationwide registry utilized overlap weighting. This approach retains the entire cohort (N = 859) and optimizes covariate balance by emphasizing clinical equipoise, effectively mimicking a randomized controlled trial. Furthermore, by employing Fine–Gray subdistribution hazard models, we appropriately addressed competing non-cancer mortality biases that frequently overestimate recurrence risks in standard Cox regressions.

Despite these strengths, inherent limitations must be acknowledged. First, due to the retrospective design, propensity score methods like overlap weighting—while robust in balancing observed variables—fundamentally cannot eliminate all biases or adjust for unmeasured confounders. Furthermore, a crucial limitation of our study design is the reliance on final pathological staging (pT2–T4) to define high-risk disease. In real-world clinical practice, the choice of surgical approach must be made preoperatively based on clinical assessments—such as cross-sectional imaging, ureteroscopy, and biopsy. However, clinical staging for UTUC is notoriously challenging and frequently underestimates the true pathological stage. Because our inclusion criteria utilized retrospective pathological confirmation, there is an inevitable discrepancy between the definitive high-risk cohort analyzed in this study and the prospective clinical reality, where surgeons must rely on inherently imperfect preoperative staging to guide their surgical decision-making. Although overlap weighting effectively mitigates baseline imbalances, residual selection bias cannot be completely excluded. As demonstrated in our unweighted cohort, patients initially selected for SUR presented with significantly more favorable disease characteristics, such as a higher proportion of smaller tumors (<2 cm) and lower pathological T stages. Consequently, these inherent baseline differences must be emphasized when interpreting the comparable oncological outcomes, as the meticulously selected nature of the SUR candidates undoubtedly contributes to their favorable long-term trajectories. Specifically, the choice of surgical approach is inherently subject to profound selection bias, driven by unrecorded clinical nuances such as precise tumor location, local anatomy, and the technical feasibility of ureteral reconstruction. This strong historical selection bias is clearly reflected in the relatively smaller unweighted SUR cohort. Second, although Fine–Gray models effectively address competing risks, they still cannot account for the “surgeon effect” or specific intraoperative technical variations. Furthermore, as our dataset spans from 1988 to 2024, it inherently encompasses significant evolutions in clinical practice. Our analysis could not explicitly adjust for the transition from open to minimally invasive or robotic surgeries, nor did it capture granular details of evolving chemotherapy protocols, such as variations in specific dosages and treatment durations across different eras. These unmeasured historical shifts in surgical and medical management could theoretically influence recurrence and metastasis patterns, introducing potential residual confounding. Third, while encompassing 21 hospitals significantly enhances generalizability within our regional demographic, this multicenter data inherently introduces institutional heterogeneity regarding surgical protocols, perioperative care, and pathological evaluations. Furthermore, the generalizability of these findings to Western populations requires careful consideration due to well-documented epidemiological differences in UTUC. In Taiwan and other Asian regions, UTUC exhibits a uniquely high incidence and an unusual female predominance, which are frequently associated with specific environmental etiologies such as aristolochic acid exposure and endemic arsenic contamination. Conversely, UTUC in Western cohorts is predominantly driven by tobacco smoking and occupational carcinogens, typically presenting with a strong male-to-female ratio. These fundamental etiological and demographic distinctions may inherently influence tumor biology, anatomical considerations during surgery (as previously noted regarding the female pelvic cavity), and overall disease trajectories. Consequently, while our findings robustly support the oncological safety of SUR in this specific cohort, caution should be exercised when extrapolating these outcomes directly to Western patients, and external validation in diverse international populations remains necessary. To definitively overcome these retrospective limitations, future large-scale, prospective randomized controlled trials (RCTs) are warranted, not only to validate these oncological outcomes but also to rigorously evaluate how SUR-mediated renal preservation translates into improved overall quality of life and enhanced tolerability for subsequent adjuvant therapies.

## 5. Conclusions

In conclusion, our multicenter study confirms that for meticulously selected patients with high-risk UTUC localized to the ureter, SUR is a safe and effective alternative to RNU. SUR provides equivalent oncological control while significantly preserving postoperative renal function. This kidney-sparing benefit is clinically crucial, ensuring patients maintain the physiological reserve required for subsequent adjuvant therapies. Ultimately, SUR represents a patient-centered surgical paradigm that successfully balances oncological safety with long-term functional preservation.

## Figures and Tables

**Table 1:** Patient Baseline Characteristics.

Variables	Levels	1 RNU	2 Segmental	*p*	SMD
		783	76		
ECOG (%)	0~1	728 (93.0)	74 (97.4)	0.22	0.206
	2~4	55 (7.0)	2 (2.6)		
Sex (%)	1 Male	328 (41.9)	41 (53.9)	0.057	0.243
	2 Female	455 (58.1)	35 (46.1)		
Age (mean (SD))		69.48 (9.50)	69.00 (9.82)	0.672	0.05
HTN (%)	0 No	324 (41.4)	27 (35.5)	0.385	0.121
	1 Yes	459 (58.6)	49 (64.5)		
UGI bleeding/ulcer (%)	0 No	730 (93.2)	71 (93.4)	1	0.008
	1 Yes	53 (6.8)	5 (6.6)		
DM (%)	0 No	563 (71.9)	45 (59.2)	0.028 *	0.27
	1 Yes	220 (28.1)	31 (40.8)		
Malignancy (%)	0 No	673 (86.0)	65 (85.5)	1	0.012
	1 Yes	110 (14.0)	11 (14.5)		
Cell Type (%)	1 Urothelial	706 (90.2)	73 (96.1)	0.139	0.234
	5 UC with variants	77 (9.8)	3 (3.9)		
Side (%)	1 Left	399 (51.0)	39 (51.3)	1	0.007
	2 Right	384 (49.0)	37 (48.7)		
Location (%)	1 Ureter	777 (99.2)	73 (96.1)	0.044 *	0.211
	2 Bladder cuff	6 (0.8)	3 (3.9)		
Multifocal (%)	0 No	710 (90.7)	72 (94.7)	0.331	0.157
	1 Yes	73 (9.3)	4 (5.3)		
Size (%)	1 <2 cm	265 (33.8)	43 (56.6)	<0.001 *	0.469
	2 ≥2 cm	518 (66.2)	33 (43.4)		
pT (%)	1 pTa/pT1	248 (31.7)	34 (44.7)	0.029 *	0.271
	2 pT2-4	535 (68.3)	42 (55.3)		
Grade (%)	1 Low grade	18 (2.3)	1 (1.3)	0.882	0.074
	2 High grade	765 (97.7)	75 (98.7)		
Pre-operation Cr level (mean (SD))		2.33 (2.55)	2.05 (1.99)	0.352	0.123
Smoking (%)	0 No	636 (81.2)	59 (77.6)	0.543	0.089
	1 Yes	147 (18.8)	17 (22.4)		
Previous Nephroureterectomy UC (%)	0 No	762 (97.3)	70 (92.1)	0.032 *	0.235
	1 Yes	21 (2.7)	6 (7.9)		
Previous bladder UC (%)	0 No	727 (92.8)	66 (86.8)	0.099	0.2
	1 Yes	56 (7.2)	10 (13.2)		
CIS (%)	0 No	605 (77.3)	66 (86.8)	0.075	0.251
	1 Yes	178 (22.7)	10 (13.2)		
Pre-operation Urine Cytology (%)	0 Negative	186 (23.8)	19 (25.0)	0.933	0.08
	1 Atypia	130 (16.6)	13 (17.1)		
	2 Positive	170 (21.7)	18 (23.7)		
	4 No cytology	297 (37.9)	26 (34.2)		
Ureteroscopy (%)	0 No	247 (31.5)	24 (31.6)	1.000	0.001
	1 Yes	536 (68.5)	52 (68.4)		
LVI (%)	0 No	650 (83.0)	65 (85.5)	0.69	0.069
	1 Yes	133 (17.0)	11 (14.5)		
Preoperative Hydronephrosis (%)	0 No	159 (20.3)	16 (21.1)	0.996	0.018
	1 Yes	624 (79.7)	60 (78.9)		
Tumor Necrosis (%)	0 No	721 (92.1)	74 (97.4)	0.148	0.238
	1 Yes	62 (7.9)	2 (2.6)		
Chemotherapy for UTUC (%)	0 No	526 (67.2)	52 (68.4)	0.926	0.027
	1 Yes	257 (32.8)	24 (31.6)		
Chemotherapy Type for UTUC (%)	0 No	526 (67.2)	53 (69.7)	0.953	0.071
	1 Neo-adjuvant	18 (2.3)	2 (2.6)		
	2 Adjuvant	176 (22.5)	15 (19.7)		
	3 Salvage/Palliative	63 (8.0)	6 (7.9)		
Chemotherapy Prescription for UTUC (%)	0 No	532 (67.9)	53 (69.7)	0.322	0.3
	1 Gemcitabine and cisplatin	87 (11.1)	10 (13.2)		
	2 MVAC	11 (1.4)	0 (0.0)		
	4 Carboplatin-based	65 (8.3)	9 (11.8)		
	5 Others	88 (11.2)	4 (5.3)		

RNU: radical nephroureterectomy; SMD: standardized mean difference. * Statistically significant (*p* < 0.05).

**Table 2:** Univariate Cox regression for overall survival (OS) and cancer-specific survival (CSS).

Variables	Levels	OS		CSS	
		HR	*p*	HR	*p*
Approach	1 RNU	1		1	
	2 Segmental	0.82 (0.53, 1.25)	0.35	1.10 (0.60, 2.04)	0.75
ECOG	0~1	1		1	
	2~4	2.54 (1.55, 4.16)	<0.001 *	2.94 (1.10, 7.86)	0.031 *
Sex	1 Male	1		1	
	2 Female	0.45 (0.29, 0.68)	<0.001 *	0.45 (0.23, 0.91)	0.026 *
Age	Mean (SD)	1.04 (1.02, 1.07)	<0.001 *	1.03 (1.00, 1.07)	0.058
HTN	0 No	1		1	
	1 Yes	1.32 (0.84, 2.09)	0.231	1.74 (0.86, 3.51)	0.126
UGI bleeding/ulcer	0 No	1		1	
	1 Yes	0.75 (0.34, 1.66)	0.475	0.34 (0.10, 1.12)	0.077
DM	0 No	1		1	
	1 Yes	1.52 (1.00, 2.29)	0.048 *	2.11 (1.09, 4.08)	0.026 *
Malignancy	0 No	1		1	
	1 Yes	1.53 (0.86, 2.74)	0.148	1.73 (0.75, 4.00)	0.199
Cell Type	1 Urothelial	1		1	
	5 UC with variants	1.62 (0.67, 3.96)	0.287	0.65 (0.25, 1.69)	0.38
Side	1 Left	1		1	
	2 Right	0.88 (0.58, 1.33)	0.535	1.04 (0.54, 2.00)	0.906
Location	1 Ureter	1		1	
	2 Bladder cuff	2.02 (0.67, 6.13)	0.214	1.71 (0.32, 9.22)	0.532
Multifocal	0 No	1		1	
	1 Yes	2.13 (1.23, 3.69)	0.007 *	1.76 (0.57, 5.38)	0.324
Size	1 <2 cm	1		1	
	2 ≥2 cm	1.06 (0.70, 1.60)	0.797	1.74 (0.87, 3.45)	0.115
pT	1 pTa/pT1	1		1	
	2 pT2-4	1.70 (1.11, 2.60)	0.015 *	2.70 (1.26, 5.81)	0.011 *
Grade	1 Low grade	1		1	
	2 High grade	0.59 (0.30, 1.16)	0.127	0.31 (0.13, 0.76)	0.011 *
Pre-operation Cr level	Mean (SD)	1.07 (0.99, 1.17)	0.096	0.94 (0.81, 1.08)	0.384
Smoking	0 No	1		1	
	1 Yes	1.62 (1.04, 2.52)	0.034 *	1.37 (0.68, 2.75)	0.378
Previous Nephroureterectomy UC	0 No	1		1	
	1 Yes	1.27 (0.52, 3.11)	0.6	0.65 (0.16, 2.61)	0.54
Previous bladder UC	0 No	1		1	
	1 Yes	1.83 (1.06, 3.15)	0.030 *	0.30 (0.09, 0.99)	0.049 *
CIS	0 No	1		1	
	1 Yes	1.18 (0.64, 2.15)	0.598	1.77 (0.77, 4.04)	0.177
Pre-operation Urine Cytology	0 Negative	1		1	
	1 Atypia	0.71 (0.38, 1.33)	0.283	0.29 (0.12, 0.72)	0.008 *
	2 Positive	1.02 (0.57, 1.83)	0.95	0.65 (0.27, 1.58)	0.344
	4 No cytology	0.79 (0.46, 1.34)	0.379	0.62 (0.28, 1.38)	0.238
LVI	0 No	1		1	
	1 Yes	1.88 (1.11, 3.16)	0.018 *	2.50 (1.17, 5.38)	0.019 *
Preoperative Hydronephrosis	0 No	1		1	
	1 Yes	0.93 (0.54, 1.60)	0.792	0.98 (0.41, 2.33)	0.969
Tumor Necrosis	0 No	1		1	
	1 Yes	0.60 (0.26, 1.37)	0.223	1.10 (0.41, 2.93)	0.847
Chemotherapy for UTUC	0 No	1		1	
	1 Yes	1.22 (0.79, 1.89)	0.376	1.83 (0.96, 3.49)	0.065
Chemotherapy Type for UTUC	0 No	1		1	
	1 Neo-adjuvant	1.51 (0.72, 3.16)	0.273	1.11 (0.24, 5.15)	0.899
	2 Adjuvant	1.12 (0.65, 1.92)	0.684	1.50 (0.69, 3.27)	0.302
	3 Salvage/Palliative	1.54 (0.78, 3.06)	0.215	3.25 (1.40, 7.52)	0.006 *
Chemotherapy Prescription for UTUC	0 No	1		1	
	1 Gemcitabine and cisplatin	1.42 (0.84, 2.41)	0.191	1.65 (0.79, 3.46)	0.186
	2 MVAC	0.30 (0.04, 2.38)	0.254	0.83 (0.10, 6.99)	0.868
	4 Carboplatin-based	1.44 (0.76, 2.70)	0.261	2.49 (1.02, 6.06)	0.05
	5 Others	0.55 (0.22, 1.43)	0.221	1.04 (0.33, 3.32)	0.949

* *p* < 0.05. OS: overall survival; CSS: cancer-specific survival; HR: hazard ratio; RNU: radical nephroureterectomy; ECOG: Eastern Cooperative Oncology Group; SD: standard deviation; HTN: hypertension; UGI: upper gastrointestinal; DM: diabetes mellitus; UC: urothelial carcinoma; pT: pathological T stage; Cr: creatinine; CIS: carcinoma in situ; LVI: lymphovascular invasion; UTUC: upper tract urothelial carcinoma; MVAC: methotrexate, vinblastine, doxorubicin, and cisplatin.

**Table 3:** Multivariate Cox regression for overall survival (OS) and cancer-specific survival (CSS).

Variables	Levels	OS		CSS	
		HR	*p*	HR	*p*
Approach	1 RNU	1		1	
	2 Segmental	0.86 (0.57, 1.29)	0.453	1.22 (0.65, 2.31)	0.536
ECOG	0~1	1		1	
	2~4	2.49 (1.37, 4.54)	0.003 *	3.76 (1.20, 11.76)	0.023 *
Sex	1 Male	1		1	
	2 Female	0.52 (0.32, 0.85)	0.009 *	0.51 (0.24, 1.09)	0.083
Age	Mean (SD)	1.05 (1.02, 1.07)	<0.001 *		
DM	0 No	1		1	
	1 Yes	1.41 (0.95, 2.09)	0.088	1.89 (0.93, 3.85)	0.079
Multifocal	0 No	1			
	1 Yes	1.89 (1.12, 3.22)	0.018 *		
pT	1 pTa/pT1	1		1	
	2 pT2-4	1.80 (1.17, 2.79)	0.008 *	2.54 (1.06, 6.07)	0.036 *
Smoking	0 No	1			
	1 Yes	1.29 (0.79, 2.10)	0.303		
Previous bladder UC	0 No	1		1	
	1 Yes	1.64 (0.89, 3.00)	0.109	0.38 (0.11, 1.29)	0.12
Pre-operation Urine Cytology	0 Negative			1	
	1 Atypia			0.25 (0.09, 0.72)	0.010 *
	2 Positive			0.75 (0.31, 1.82)	0.519
	4 No cytology			0.69 (0.31, 1.54)	0.369
LVI	0 No	1		1	
	1 Yes	1.57 (0.93, 2.64)	0.092	2.21 (1.05, 4.63)	0.036 *
Chemotherapy Type for UTUC	0 No			1	
	1 Neo-adjuvant			1.04 (0.12, 9.17)	0.971
	2 Adjuvant			0.86 (0.36, 2.04)	0.733
	3 Salvage/Palliative			2.84 (1.25, 6.47)	0.013 *

* *p* < 0.05. OS: overall survival; CSS: cancer-specific survival; HR: hazard ratio; RNU: radical nephroureterectomy; ECOG: Eastern Cooperative Oncology Group; SD: standard deviation; DM: diabetes mellitus; pT: pathological T stage; UC: urothelial carcinoma; LVI: lymphovascular invasion; UTUC: upper tract urothelial carcinoma.

**Table 4:** Multivariate Fine–Grey Hazard Model for Cancer-Specific Survival (CSS), Metastasis-Free Survival (MFS) and Local Recurrence-Free Survival (LRFS).

Variables	Levels	CSS		MFS		LRFS	
		HR	*p*	HR	*p*	HR	*p*
Approach	1 RNU	1		1		1	
	2 Segmental	0.94 (0.52, 1.70)	0.835	0.88 (0.48, 1.59)	0.664	0.74 (0.39, 1.40)	0.351
Sex	1 Male	1		1		1	
	2 Female	0.74 (0.49, 1.09)	0.129	0.80 (0.54, 1.19)	0.269	0.58 (0.41, 0.81)	0.001 *
Malignancy	0 No	1					
	1 Yes	1.56 (1.02, 2.40)	0.041 *				
Smoking	0 No	1		1			
	1 Yes	1.31 (0.84, 2.04)	0.239	1.43 (0.93, 2.21)	0.106		
Previous bladder UC	0 No	1					
	1 Yes	0.31 (0.11, 0.84)	0.022 *				

* *p* < 0.05. CSS: cancer-specific survival; MFS: metastasis-free survival; LRFS: local recurrence-free survival; HR: hazard ratio; RNU: radical nephroureterectomy; UC: urothelial carcinoma.

**Table 5:** Univariate Cox Regression for Metastasis-Free Survival (MFS) and Local Recurrence-Free Survival (LRFS).

Variables	Levels	MFS		LRFS	
		HR	*p*	HR	*p*
Approach	1 RNU	1		1	
	2 Segmental	0.92 (0.50, 1.70)	0.796	2.47 (1.15, 5.31)	0.020 *
ECOG	0~1	1		1	
	2~4	0.54 (0.17, 1.72)	0.293	0.48 (0.09, 2.76)	0.414
Sex	1 Male	1		1	
	2 Female	0.33 (0.18, 0.62)	<0.001 *	0.36 (0.13, 0.98)	0.046 *
Age	Mean (SD)	1.00 (0.97, 1.03)	0.887	1.04 (0.99, 1.08)	0.1
HTN	0 No	1		1	
	1 Yes	1.29 (0.66, 2.50)	0.453	3.77 (1.18, 12.05)	0.025 *
UGI bleeding/ulcer	0 No	1		1	
	1 Yes	0.33 (0.10, 1.07)	0.064	1.37 (0.29, 6.50)	0.69
DM	0 No	1		1	
	1 Yes	1.62 (0.87, 3.02)	0.129	2.05 (0.81, 5.18)	0.13
Malignancy	0 No	1		1	
	1 Yes	1.54 (0.63, 3.76)	0.348	1.55 (0.48, 5.00)	0.46
Cell Type	1 Urothelial	1		1	
	5 UC with variants	0.72 (0.25, 2.03)	0.532	0.28 (0.07, 1.08)	0.065
Side	1 Left	1		1	
	2 Right	1.46 (0.79, 2.71)	0.223	0.98 (0.38, 2.49)	0.96
Location	1 Ureter	1		1	
	2 Bladder cuff	2.03 (0.51, 8.07)	0.316	-	
Multifocal	0 No	1		1	
	1 Yes	1.54 (0.48, 4.96)	0.468	0.15 (0.03, 0.76)	0.022 *
Size	1 <2cm	1		1	
	2 ≥2 cm	1.52 (0.81, 2.83)	0.189	2.17 (0.86, 5.47)	0.102
pT	1 pTa/pT1	1		1	
	2 pT2-4	2.32 (1.16, 4.64)	0.017 *	2.59 (1.04, 6.44)	0.041 *
Grade	1 Low grade	1		1	
	2 High grade	12.91 (1.32, 126.35)	0.028 *	2.56 (0.25, 25.81)	0.427
Pre-operation Cr level	Mean (SD)	0.88 (0.71, 1.08)	0.222	1.19 (1.02, 1.38)	0.030 *
Smoking	0 No	1		1	
	1 Yes	1.86 (1.01, 3.43)	0.045 *	2.52 (1.01, 6.31)	0.048 *
Previous Nephroureterectomy UC	0 No	1		1	
	1 Yes	1.29 (0.37, 4.47)	0.687	2.26 (0.69, 7.39)	0.177
Previous bladder UC	0 No	1		1	
	1 Yes	0.58 (0.22, 1.50)	0.259	0.56 (0.14, 2.25)	0.416
CIS	0 No	1		1	
	1 Yes	1.04 (0.36, 2.97)	0.946	3.17 (1.00, 10.08)	0.05
Pre-operation Urine Cytology	0 Negative	1		1	
	1 Atypia	0.54 (0.22, 1.38)	0.199	0.33 (0.06, 1.65)	0.175
	2 Positive	0.71 (0.28, 1.76)	0.454	0.44 (0.10, 1.89)	0.268
	4 No cytology	1.00 (0.49, 2.06)	0.996	0.76 (0.27, 2.19)	0.616
LVI	0 No	1		1	
	1 Yes	3.38 (1.61, 7.09)	0.001 *	1.59 (0.38, 6.59)	0.521
Preoperative Hydronephrosis	0 No	1		1	
	1 Yes	1.16 (0.51, 2.64)	0.72	1.55 (0.43, 5.62)	0.505
Tumor Necrosis	0 No	1		1	
	1 Yes	1.68 (0.72, 3.96)	0.233	1.07 (0.33, 3.47)	0.905
Chemotherapy for UTUC	0 No	1		1	
	1 Yes	3.97 (2.09, 7.55)	<0.001 *	1.58 (0.62, 4.00)	0.338
Chemotherapy Type for UTUC	0 No	1		1	
	1 Neo-adjuvant	1.67 (0.34, 8.15)	0.523	0.44 (0.05, 4.31)	0.483
	2 Adjuvant	2.82 (1.27, 6.25)	0.011 *	0.82 (0.19, 3.62)	0.796
	3 Salvage/Palliative	7.30 (3.63, 14.67)	<0.001 *	3.64 (1.45, 9.12)	0.006 *
Chemotherapy Prescription for UTUC	0 No	1		1	
	1 Gemcitabine and cisplatin	4.09 (2.04, 8.21)	<0.001 *	1.97 (0.58, 6.70)	0.279
	2 MVAC	1.81 (0.22, 15.30)	0.584	0.01 (0.00, 0.02)	<0.001 *
	4 Carboplatin-based	4.54 (1.94, 10.64)	<0.001 *	2.43 (0.87, 6.78)	0.088
	5 Others	2.69 (0.95, 7.63)	0.062	0.22 (0.05, 0.91)	0.036 *

* *p* < 0.05. MFS: metastasis-free survival; LRFS: local recurrence-free survival; HR: hazard ratio; RNU: radical nephroureterectomy; ECOG: Eastern Cooperative Oncology Group; SD: standard deviation; HTN: hypertension; UGI: upper gastrointestinal; DM: diabetes mellitus; UC: urothelial carcinoma; pT: pathological T stage; Cr: creatinine; CIS: carcinoma in situ; LVI: lymphovascular invasion; UTUC: upper tract urothelial carcinoma; MVAC: methotrexate, vinblastine, doxorubicin, and cisplatin.

**Table 6:** Multivariate Cox Regression for Metastasis-Free Survival (MFS) and Local Recurrence-Free Survival (LRFS).

Variables	Levels	MFS		LRFS	
		HR	*p*	HR	*p*
Approach	1 RNU	1		1	
	2 Segmental	0.97 (0.53, 1.74)	0.909	4.34 (1.60, 11.77)	0.004 *
Sex	1 Male	1		1	
	2 Female	0.52 (0.24, 1.11)	0.091	0.48 (0.16, 1.45)	0.193
Age	Mean (SD)				
HTN	0 No			1	
	1 Yes			3.25 (1.14, 9.26)	0.028 *
Location	1 Ureter				
	2 Bladder cuff				
Size	1 <2 cm				
	2 ≥2 cm				
pT	1 pTa/pT1	1		1	
	2 pT2-4	1.66 (0.82, 3.34)	0.157	3.57 (1.35, 9.40)	0.010 *
Grade	1 Low grade	1			
	2 High grade	11.35 (1.18, 108.99)	0.035 *		
Pre-operation Cr level	Mean (SD)			1.37 (1.17, 1.60)	<0.001 *
Smoking	0 No	1		1	
	1 Yes	1.14 (0.54, 2.41)	0.729	2.08 (0.63, 6.91)	0.231
Previous bladder UC	0 No				
	1 Yes				
CIS	0 No				
	1 Yes				
Pre-operation Urine Cytology	0 Negative				
	1 Atypia				
	2 Positive				
	4 No cytology				
LVI	0 No	1			
	1 Yes	3.65 (1.60, 8.35)	0.002 *		
Chemotherapy Type for UTUC	0 No	1		1	
	1 Neo-adjuvant	8.34 (0.57, 122.16)	0.121	0.08 (0.00, 1.54)	0.095
	2 Adjuvant	10.63 (2.26, 50.04)	0.003 *	0.21 (0.04, 1.23)	0.084
	3 Salvage/Palliative	48.61 (10.52, 224.74)	<0.001 *	0.76 (0.13, 4.47)	0.761
Chemotherapy Prescription for UTUC	0 No			1	
	1 Gemcitabine and cisplatin			4.41 (0.73, 26.80)	0.107
	2 MVAC			-	
	4 Carboplatin-based			5.53 (1.07, 28.66)	0.042 *
	5 Others			0.62 (0.15, 2.63)	0.518

* *p* < 0.05. MFS: metastasis-free survival; LRFS: local recurrence-free survival; HR: hazard ratio; RNU: radical nephroureterectomy; SD: standard deviation; HTN: hypertension; UC: urothelial carcinoma; pT: pathological T stage; Cr: creatinine; CIS: carcinoma in situ; LVI: lymphovascular invasion; UTUC: upper tract urothelial carcinoma; MVAC: methotrexate, vinblastine, doxorubicin, and cisplatin.

**Table 7:** Outcome variables (overlap-weighted).

Variables	Levels	Unweighted					Overlap-Weighted		
		1 RNU	2 Segmental	*p*	SMD	1 RNU	2 Segmental	*p*	SMD
N		783	76			429.5	429.5		
Clavien–Dindo Classification (%)	0 No Complication	480 (61.3)	48 (63.2)	0.284	0.175	277.0 (64.5)	269.7 (62.8)	0.399	0.163
	1 Grade I/II	269 (34.4)	22 (28.9)			133.9 (31.2)	124.2 (28.9)		
	2 Grade III/IV	34 (4.3)	6 (7.9)			18.7 (4.4)	35.5 (8.3)		
Surgical Margin (%)	0 Free	741 (94.6)	70 (92.1)	0.512	0.102				
	1 Positive	42 (5.4)	6 (7.9)						
Post-operation Bladder UC (%)	0 No	579 (73.9)	46 (60.5)	0.018 *	0.289	306.4 (71.3)	264.5 (61.6)	0.095	0.208
	1 Yes	204 (26.1)	30 (39.5)			123.1 (28.7)	165.0 (38.4)		
Local Recurrence (%)	0 No	743 (94.9)	66 (86.8)	0.009 *	0.282	409.7 (95.4)	373.0 (86.8)	0.004 *	0.304
	1 Yes	40 (5.1)	10 (13.2)			19.8 (4.6)	56.5 (13.2)		
Lymphatic Metastasis (%)	0 No	693 (88.5)	68 (89.5)	0.949	0.031	387.3 (90.2)	388.9 (90.5)	0.917	0.013
	1 Yes	90 (11.5)	8 (10.5)			42.2 (9.8)	40.6 (9.5)		
Distant Metastasis (%)	0 No	655 (83.7)	64 (84.2)	1	0.015	361.7 (84.2)	360.0 (83.8)	0.932	0.011
	1 Yes	128 (16.3)	12 (15.8)			67.8 (15.8)	69.5 (16.2)		
Overall Mortality (%)	0 No	480 (61.3)	51 (67.1)	0.384	0.121	266.4 (62.0)	283.6 (66.0)	0.514	0.084
	1 Yes	303 (38.7)	25 (32.9)			163.1 (38.0)	145.9 (34.0)		
Cancer Specific (%)	0 No	659 (84.2)	64 (84.2)	1	0.001	370.5 (86.3)	358.2 (83.4)	0.519	0.08
	1 Yes	124 (15.8)	12 (15.8)			59.0 (13.7)	71.3 (16.6)		
Intravesical Recurrence (%)	0 No	651 (83.1)	66 (86.8)	0.505	0.104	342.6 (79.8)	376.6 (87.7)	0.109	0.215
	1 Yes	132 (16.9)	10 (13.2)			86.9 (20.2)	52.9 (12.3)		
Follow-up OS/CSS (mean (SD))		57.50 (41.55)	63.79 (42.24)	0.209	0.15	57.87 (40.39)	64.08 (43.33)	0.256	0.148
Follow-up BRFS/DFS (mean (SD))		42.21 (34.45)	46.48 (36.97)	0.306	0.12	41.98 (33.34)	46.48 (37.60)	0.336	0.127
Pre-operation eGFR (mean (SD))		46.70 (25.96)	46.78 (22.91)	0.98	0.003	49.91 (25.52)	46.49 (23.13)	0.247	0.141
Post-operation eGFR 1 month		38.83 (22.62)	46.54 (25.26)	0.005 *	0.321	39.33 (21.71)	46.38 (25.08)	0.022 *	0.301
Last eGFR (mean (SD))		37.67 (24.85)	41.05 (24.19)	0.257	0.138	37.42 (24.08)	41.16 (24.24)	0.217	0.155
Delta eGFR in 1 month		−7.87 (18.99)	−0.24 (16.37)	0.001 *	0.43	−10.58 (19.10)	−0.11 (15.44)	<0.001 *	0.603
Delta eGFR last (mean (SD))		−9.04 (21.45)	−5.73 (16.98)	0.193	0.171	−12.49 (21.47)	−5.33 (17.04)	0.001 *	0.369

* Statistically significant (*p* < 0.05). RNU: radical nephroureterectomy; eGFR: estimated glomerular filtration rate; SD: standard deviation.

## Data Availability

The data presented in this study are not publicly available due to strict patient privacy and ethical restrictions associated with the Taiwan Upper Tract Urothelial Carcinoma Collaboration database. However, the data are available on reasonable request from the corresponding authors, subject to appropriate institutional review board approvals.

## Supplementary Materials

The following supporting information can be downloaded at: https://www.mdpi.com/article/10.3390/cancers18152527/s1. Table S1. Preoperative and Postoperative Renal Function Dynamics and Cisplatin Eligibility.

## Abbreviations

The following abbreviations are used in this manuscript:

CIS	carcinoma in situ
Cr	creatinine
CSS	cancer-specific survival
DFS	disease-free survival
DM	diabetes mellitus
ECOG	Eastern Cooperative Oncology Group
eGFR	estimated glomerular filtration rate
HR	hazard ratio
HTN	hypertension
IVRFS	intravesical recurrence-free survival
LRFS	local recurrence-free survival
LVI	lymphovascular invasion
MFS	metastasis-free survival
MVAC	methotrexate, vinblastine, doxorubicin, and cisplatin
OS	overall survival
pT	pathological T stage
RNU	radical nephroureterectomy
SD	standard deviation
SMD	standardized mean difference
SUR	segmental ureteral resection
UC	urothelial carcinoma
UGI	upper gastrointestinal
UTUC	upper tract urothelial carcinoma
