# Supplementary material for: Feasibility and Oncological Outcomes of Segmental Ureteral Resection Versus Radical Nephroureterectomy for High-Risk Ureteral Urothelial Carcinoma
Source: Cancers (Basel). 2026 Aug 6;18(15):2527. doi: 10.3390/cancers18152527 (PMC13464848; doi:10.3390/cancers18152527)
Supplement: Supplementary file 1 [file cancers-18-02527-s001.zip › cancers-4434010-supplementary.pdf]

Supplementary Materials

Table S1. Preoperative and Postoperative Renal Function Dynamics and Cisplatin Eligibility.

Variables	Levels	1 RNU	2 SUR	P	SMD
N		783	76		
Pre-operative eGFR (%)	1 Stage 1	44 (5.6)	3 (3.9)	0.505	0.216
	2 Stage 2	173 (22.1)	17 (22.4)		
	3 Stage 3	373 (47.6)	37 (48.7)		
	4 Stage 4	73 (9.3)	11 (14.5)		
	5 Stage 5	120 (15.3)	8 (10.5)		
Post-operative 1 month eGFR (%)	1 Stage 1	8 (1.0)	7 (9.2)	**<0.001***	0.410
	2 Stage 2	121 (15.5)	12 (15.8)		
	3 Stage 3	373 (47.6)	36 (47.4)		
	4 Stage 4	124 (15.8)	11 (14.5)		
	5 Stage 5	157 (20.1)	10 (13.2)		
Pre-operative (%)	eGFR>60	217 (27.7)	20 (26.3)	0.900	0.031
Post-operative (%)	eGFR >60	129 (16.5)	19 (25.0)	0.085	0.211

eGFR = estimated glomerular filtration rate; RNU = radical nephroureterectomy; SUR = segmental ureteral resection; SMD = standardized mean difference. eGFR stages correspond to chronic kidney disease (CKD) stages 1 through 5. An eGFR threshold of > 60 mL/min/1.73m^2 is conventionally utilized to define theoretical eligibility for optimal full-dose cisplatin-based adjuvant chemotherapy.

* *Statistically significant (P < 0.05).*
